# A New Dataset for Source Identification of High Dynamic Range Images

**DOI:** 10.3390/s18113801

**Published:** 2018-11-06

**Authors:** Omar Al Shaya, Pengpeng Yang, Rongrong Ni, Yao Zhao, Alessandro Piva

**Affiliations:** 1 Department of Information Engineering, University of Florence, Via di S. Marta, 3, 50139 Florence, Italy; omar.alshaya@unifi.it; 2 Department of Electronic Media, Saudi Electronic University, Abi Bakr As Sadiq Rd, Riyadh 11673, Saudi Arabia; 3 Beijing Key Laboratory of Advanced Information Science and Network Technology, Beijing Jiaotong University, Beijing 100044, China; ppyang@bjtu.edu.cn (P.Y.); yzhao@bjtu.edu.cn (Y.Z.); 4 Institute of Information Science, Beijing Jiaotong University, Beijing 100044, China; 5 FORLAB, Multimedia Forensics Laboratory, PIN Scrl, Piazza G. Ciardi, 25, 59100 Prato, Italy

**Keywords:** dataset, multimedia forensics, image forensics, HDR, source identification

## Abstract

Digital source identification is one of the most important problems in the field of multimedia forensics. While Standard Dynamic Range (SDR) images are commonly analyzed, High Dynamic Range (HDR) images are a less common research subject, which leaves space for further analysis. In this paper, we present a novel database of HDR and SDR images captured in different conditions, including various capturing motions, scenes and devices. As a possible application of this dataset, the performance of the well-known reference pattern noise-based source identification algorithm was tested on both kinds of images. Results have shown difficulties in source identification conducted on HDR images, due to their complexity and wider dynamic range. It is concluded that capturing conditions and devices themselves can have an impact on source identification, thus leaving space for more research in this field.

## 1. Introduction

**Figure 1:**
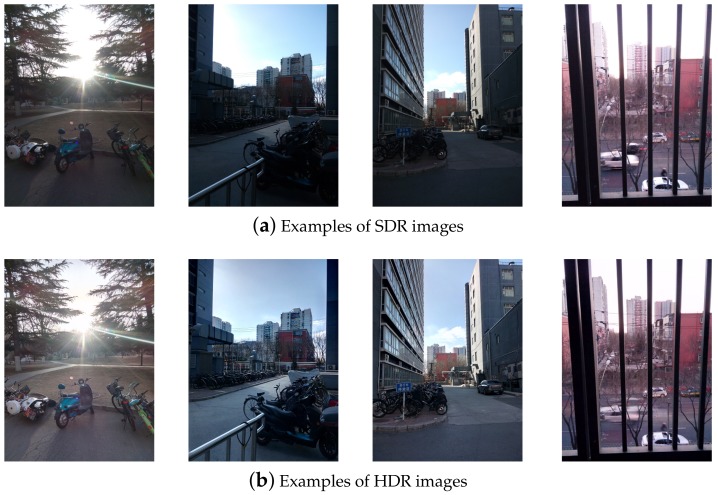
Examples of (**a**) Standard Dynamic Range (SDR) and (**b**) High Dynamic Range (HDR) images.

Digital media have become a crucial source of information worldwide. The increase in popularity of smartphone devices, camcorders, cameras and other digital media devices has brought many advantages, but the security aspects are endangered. The obtained data can easily be transferred and edited to change their perspective altogether. This phenomenon has led to difficulties pertaining to the authentication of the information shared in the form of multimedia content. Multimedia forensics is a branch of forensic sciences that deals with this problem. Its role ranges from the investigation of operational problems to the recovery of intentionally or unintentionally caused damage of the original information [1,2]. One of the cornerstones of this branch is accumulation of and fetching data regarding criminal activities [3,4], sharing of the data that have been tampered with and content manipulation [5]. The main problem is that alterations and manipulations can be performed at such a high level that it becomes very difficult to distinguish the original content from the one that has been tampered with or is fake. Authentication of the information involves tracing of specific codes, links, logos, ambiance, lighting or any sort of clue that was present when the original content was made. Identifying the source of the information can therefore be of high importance. Numerous algorithms have been developed to perform digital image source identification. The process can be conducted using approaches such as artifact detection [6,7], detection of pixel defects [8] and supervised learning [9]. Source identification based on detection of the reference pattern noise [10,11], better known as Photo-Response Non-Uniformity noise (PRNU), has proven to be very successful. It was first introduced in [12] as a general solution for reliable source identification. This approach is based on the fact that pixels have different sensitivity to illumination, which provides the ability to recognize the source device, even if the manufacturer did not imprint an invisible watermark on the images. Despite the significant success of the PRNU method, there is still space for further research. Image capturing devices have developed rapidly in the past decade, providing a wide range of options, such as image stitching [13] enabled by multi-lense mobile devices [14], various techniques of image composition [15,16] and fusing. Smartphone devices are commonly equipped to provide not only image stitching, but also a wide range of image post-processing options that the user can apply without knowing which processes are performed to get the final result. The High Dynamic Range (HDR) is a very popular option, which provides the ability of representing a wider luminance range, in comparison to the conventional Standard Dynamic Range (SDR) images, and generate much more realistic visual content [17], as shown in Figure 1. While the SDR profile does not allow big luminosity adjustments and is therefore sensitive in cases of bad lighting conditions and facing the source of light, the HDR profile copes with these problems and simulates the way the human visual system adjusts to these kinds of lighting changes. This can be noticed by comparing the images given in Figure 1. HDR images are believed to become important multimedia files in the near future. As their possibilities are still a common research topic, new standards have been created for still HDR images [17], and a number of researchers in the field of multimedia security have studied steganography [18] and watermarking [19] for this image type. However, as far as we know, no research or dataset focusing on source camera identification based on HDR images captured by smartphone devices is available up to this date. This fact gave us a strong motivation to build a novel image dataset.

In contrast to the SDR images, their HDR counterparts are characterized by a high irradiance dynamic range and localized contrast [20,21]. Currently, there are three major tools for generating HDR images: Computer Graphics (CG), HDR cameras and SDR cameras. As smartphone devices are commonly used for HDR image acquisition using ‘HDR MODE’, they are worth being paid attention. In general, the acquisition [22,23,24,25] includes several important stages: multi-exposure image capturing [23], image alignment [26], image fusion [22,23,24,27] and tone mapping [28,29]. During the stage of image alignment, geometric transformation can be executed on the misaligned images. Furthermore, image fusion and tone mapping can lead to a non-linear transformation of pixel values. Considering the previous statements, it can be concluded that the PRNU-based method, an effective way to identify the source camera for SDR images, is facing new challenges in the case of HDR images. Therefore, it is of high importance to test the performance of the PRNU-based method on HDR images.

This paper highlights a forensics analysis of HDR images deploying the novel datasets acquired through mobile camera applications. It caters to the authentication techniques of forensic analysis through the source identification process and forgery detection. This will be profitable in backtracking the rightful owner of the content and will be helpful in determination of the information’s authenticity. Moreover, forgery detection will analyze the amount of tampering with or manipulation done to the original information, as well as techniques or processes through which it has been performed.

The remainder of paper is organized as follows: Section 2 gives the dataset description. The principles of PRNU detection are briefly introduced in Section 3. Section 4 and Section 5 present the performed experiments and their results, respectively. Finally, Section 6 gives the conclusions to this research.

## 2. The Dataset

Following the procedure adopted to build the VISION Dataset [30], a novel dataset with compressed HDR images was created. The term CDR (Compressed Dynamic Range), which describes compressed HDR images, is not commonly used in the literature, but is often referred to as HDR. Therefore, we adopt the term HDR in the remainder of this paper. Twenty three mobile devices were used for capturing a total of 5415 images in different scenarios. All images described in this paper will be available at https://lesc.dinfo.unifi.it/en/datasets. This approach enabled the analysis of differences between HDR and SDR images, their application and usability in source identification.

The brands of the employed devices included Huawei, Samsung, Xiaomi, Gionee, One Plus, Asus and Apple. Among them, there were seven different models of Huawei, four of Samsung, three of Xiaomi, six of Apple and one for each of Asus, Gionee and OnePlus. Seventeen of the employed devices used the Android operating system, while six of them used iOS. Further information about the devices, the image resolutions they provide and the number of images taken is given in Table 1. Devices were named in accordance with their operating system, e.g., “A” stands for a device that uses Android, while “I” indicates the iOS operating system. Images were further named in the format “device_category_movement_number”, where “device” represents the abbreviated name of the device model, as previously explained, “category” refers to HDR or SDR, “movement” defines camera movements during the acquisition, which can be TRIPOD, HAND or SHAKING, while “number” represents the ordinal number of the captured image. All the selected mobile devices were configured to capture photos in the default camera settings of the software system. Photos were taken without using flash, in different atmospheres, including both indoor and outdoor scenes. As the analysis requires both HDR and standard SDR images of the same scene, two photos of each scene were captured.

For source identification purposes, the images were divided into two categories: FLAT and NAT. FLAT images represent approximately uniform surfaces, which are flattish in terms of texture and allow computing a clearer PRNU reference, in comparison to the images representing natural scenes. Thus, FLAT images are devoted to sensor-noise-based source identification. Specifically, images of walls and skies are in this category. On the other hand, NAT images are available for any image forensic application. While FLAT images are homogeneous, NAT images can contain a large number of details and colors. Therefore, the NAT category includes generic images, which contain a large span of scenes. Depending on the way they were created, NAT images were further divided into three categories:

- images taken from the tripod (TRIPOD),
- images taken by hand (HAND),
- images taken by a shaky hand (SHAKING).

**Figure 2:**
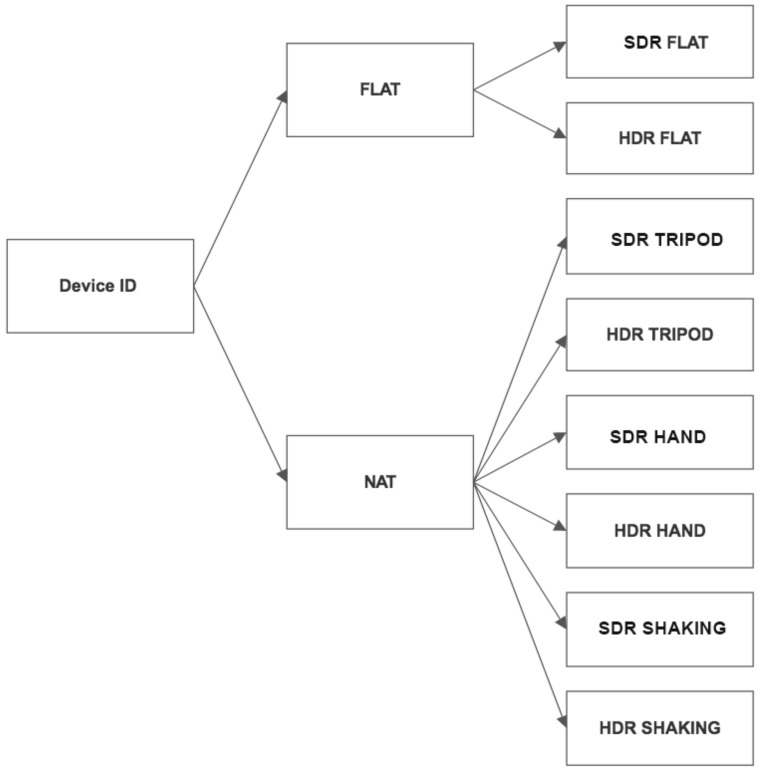
The dataset structure.

**Figure 3:**
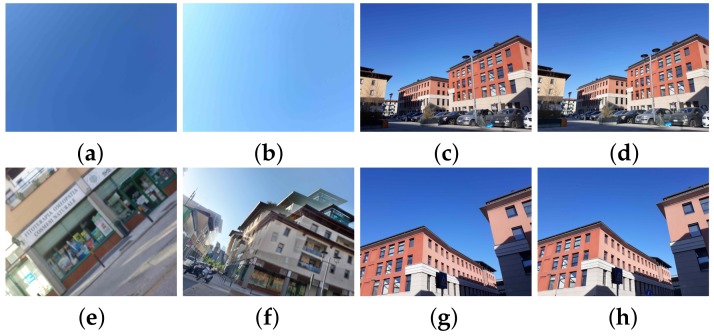
Sample pictures from the dataset: (**a**) FLAT SDR, (**b**) FLAT HDR, (**c**) Tripod SDR, (**d**) Tripod HDR, (**e**) Shaky hand SDR, (**f**) Shaky hand HDR, (**g**) Hand SDR and (**h**) Hand HDR.

The stability of the image highly depends on the camera steadiness, which was the reason for capturing the images with three different motions. Tripod allows the camera to be as still as possible. Capturing the image with the device held in a steady hand is the most common way of taking photographs, which usually causes small pixel artifacts that are not very noticeable to the human eye. Finally, images taken by a shaky hand can be blurred, because of the pixel shifting, caused by the camera shaking. As HDR images are usually obtained from multiple SDR images, it is expected that the motion could have an impact on the source identification results for HDR images. The previously described structure of the dataset is shown in Figure 2. A sample of images from the created dataset is given in Figure 3.

## 3. PRNU-Based Source Identification

PRNU is also known as a unique stochastic fingerprint of imaging sensors, and it is obtained from a set of *N* images taken by the same device, using the Maximum Likelihood Estimator (MLE) [11]. It is shown that the best estimation performances can be achieved if the number of images *N* is a sufficiently large integer and the images are uniformly white, but not fully saturated [11]. In this paper, an improved PRNU estimator, presented in [11], is employed. The improvement is primarily reflected in the reduced number of images (minimum of 30 instead of 50 images) required for PRNU estimation, retaining the basic concepts of the original method.

MLE is modeled from the simplified sensor output model [11], defined by Equation (1), which applies to each pixel of the image. Symbol *I* denotes the luminance value of the analyzed pixel; *Y* is illumination; *g* stands for the channel color gain; γ is the correction factor; $\Theta_{q}$ is the quantization noise; while Λ includes a combination of other noise sources [31]. Finally, *K* is the PRNU factor, which is a noise-like signal responsible for the fingerprint [11] and which is estimated from *N* images taken by the camera.

(1) $$I=g^{\gamma }\times {\left[(1+K)Y+\Lambda \right]}^{\gamma }+\Theta_{q}$$

**Figure 4:**
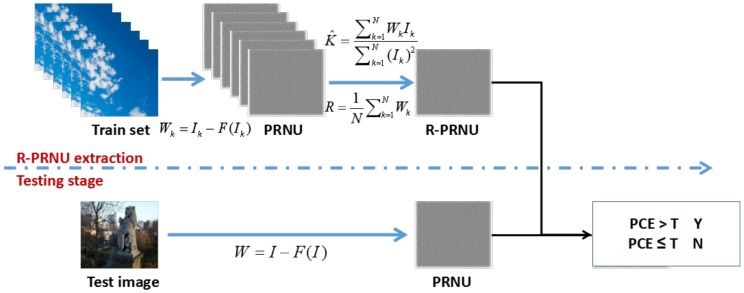
The framework of the Photo Response Non-Uniformity (PRNU)-based algorithm. PCE, Peak to Correlation Energy ratio.

The fingerprint is obtained as an approximation to the Photo Response Non-Uniformity (PRNU) noise [12]. The framework of the PRNU-based algorithm is shown in Figure 4. *N* images from the set are first denoised using the wavelet-based denoising filter. Noise residuals *W* are then averaged in order to compute the fingerprint. In particular, the maximum likelihood estimate $\hat{K}$ is obtained from partial derivation of the log-likelihood L(K) of ratio $\frac{W}{I}$ solved for *K* [11], as shown in Equation (2), where $\sigma^{2}$ denotes the variance of White Gaussian Noise (WGN). WGN is accepted as a simplified model of the noise term, without significant impact on the results of the procedure.

(2) $$\frac{\delta L(K)}{\delta K}=\sum_{k=1}^{N}\frac{W_{k}/I_{k}-K}{\sigma^{2}/{\left(I_{k}\right)}^{2}}=0\Rightarrow \hat{K}=\frac{\sum_{k=1}^{N}W_{k}I_{k}}{\sum_{k=1}^{N}{\left(I_{k}\right)}^{2}}$$

In order to perform a source identification, the noise is extracted from the image under analysis and then correlated with the previously found camera reference pattern noise (fingerprint). The maximum of the normalized correlation ρ is considered to be a good approximation of the generalized likelihood ratio test [32] and is therefore computed, in accordance with the statistical signal theory relation for the correlation computation. Finally, the Neyman–Pearson approach can be employed for correlation thresholding and final source identification.

Due to the dependence of the correlation factor on the image size, it is not a suitable parameter for further analysis of the results. The Peak to Correlation Energy ratio (PCE) is a better comparison factor [33], and it can be defined by Equation (3), where $s_{peak}$ denotes the coordinates of the peak, *m* and *n* are the image dimensions and *M* is a small neighborhood around the peak [33].

(3) $$\mathrm{PCE}=\frac{\rho {\left(s_{peak};X,Y\right)}^{2}}{\frac{1}{mn-|M|}\sum_{s\notin M}^{}\rho {\left(s;X,Y\right)}^{2}}$$

PCE considers a possible special shifts between the fingerprint and the noise extracted from the image due to possible cropping or use of the image. Then, a correlation is conducted for each shift, and if correlation proof is found, the corresponding shift is considered to give the correct output.

## 4. Experiments

The experiment was conducted by computing a camera fingerprint over three different sets of flat images, for each employed device, namely:

- HDR, which contained a set of 50–87 flat HDR images per device,
- SDR, which contained a set of 50–59 flat SDR images per device,
- MIX, which contained a set of 100–137 images, including both flat HDR and flat SDR images per device.

Each fingerprint was used for further computation of the correlation with the noise extracted from each image belonging to one of the natural datasets.

After performing the PCE computation for all of the images taken by the device of interest, plots of PCE values for single images were generated for each of the three analyzed categories. These results served as a starting point of digital source identification reliability analysis. If the PCE value of an image was above the threshold, the image was considered to be reliably paired with a digital source with which it was captured. The threshold value was chosen to be 45, in accordance with the conclusions drawn in [11].

## 5. Results

The analysis was first conducted by comparison of the PCE values of single SDR and HDR images when the noise extracted from them was correlated with fingerprints created from flat SDR, HDR and MIX sets of images. The impact of image and fingerprint types, as well as the impact of motions that occurred during image capturing were observed. The reliability of source identification was examined afterwards.

It is worth noting that PCE computation was performed for all the analyzed images captured by a certain camera model and was subsequently averaged. Results have shown that averaged PCE was less prone to result variations, and camera movements had less impact on the results in the case of using this parameter.

### 5.1. Analysis in Terms of Image Type: SDR vs. HDR

**Figure 5:**
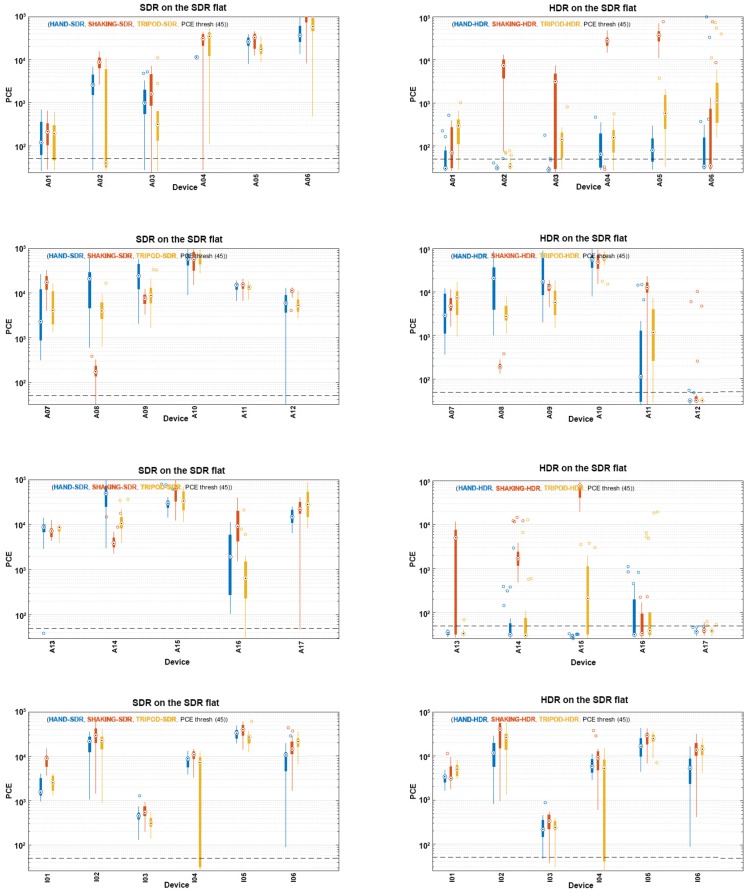
PCE values obtained for SDR and HDR images when compared with a flat SDR-based fingerprint.

First of all, we analyzed the correlation of images with the flat SDR-based fingerprint. As expected, generally higher PCE values were obtained using SDR images and a flat SDR-based fingerprint, considering they were of the same type. The results can be seen in Figure 5. Devices A01–A06 have shown the biggest difference between PCE values in the case when the captures were taken using a tripod. While SDRs were characterized by high PCE values in that case, the PCEs of most HDRs were low and sometimes were even below the threshold. The difference in terms of a higher PCE value for SDRs in comparison to HDRs was noticeable in the case of captures taken using a tripod, as well. In the case of captures made by a shaky hand, an analogy to the previous two cases cannot be applied. While Devices A02, A04 and A05 were shown to have similar PCE values for both SDR and HDR images, the other half of the devices were shown to have higher PCE values when SDR images were employed in combination with the SDR-based fingerprint.

Similar results were obtained with Devices A07–A17. The differences between PCE values of SDR and HDR images were not as emphasized as for the previously analyzed set of devices, rather similar in the case of Devices A07–A10. On the other hand, A11–A17 followed the same behavior as A01–A06. Images captured by a shaky hand did not follow any pattern. While PCEs were similar for Devices A08–A11 and A15, they were distinctively higher for SDR than HDR images captured by other devices from the mentioned set.

Finally, an analysis of Devices I01–I06 showed similar PCE values for both image types, regardless of the camera motion. While PCE values obtained by analyzing SDR images from Devices I02–I06 were slightly higher than the ones corresponding to the HDR images, I01 showed unexpected results. Indeed, with this device, the PCE values for images taken by a steady and by a shaky hand were shown to be higher for HDR images correlated with the SDR-based fingerprint.

**Figure 6:**
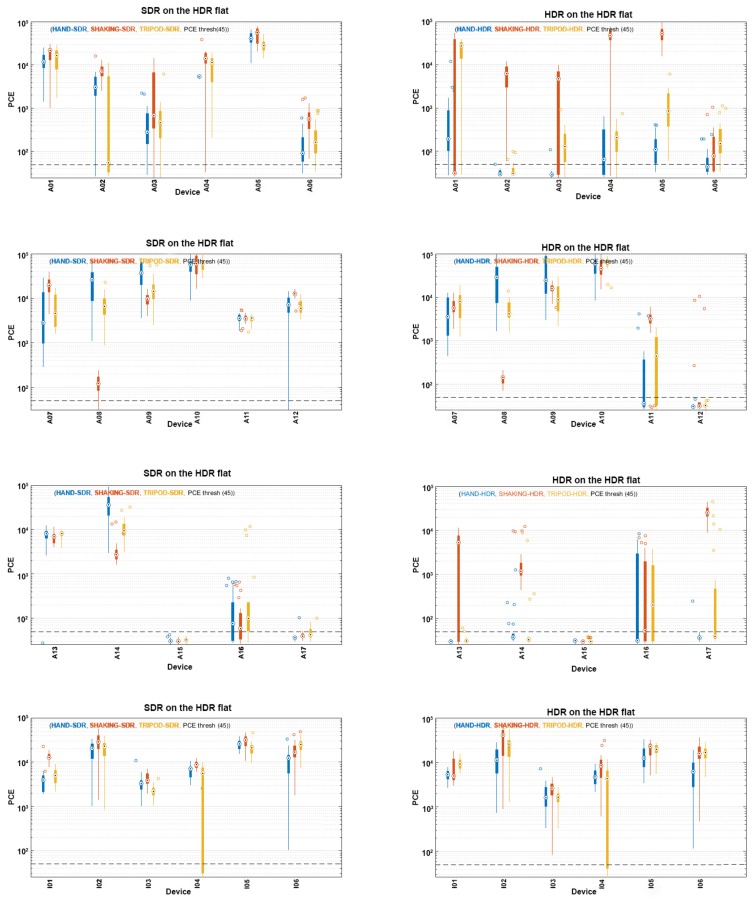
PCE values obtained by SDR and HDR images when compared with a flat HDR-based fingerprint.

The analysis was further conducted by comparison of the PCE values when noise from the SDR and HDR images was correlated with the HDR-based fingerprint. The results are shown in Figure 6. All the devices showed analogous behavior as the previously described one.

**Figure 7:**
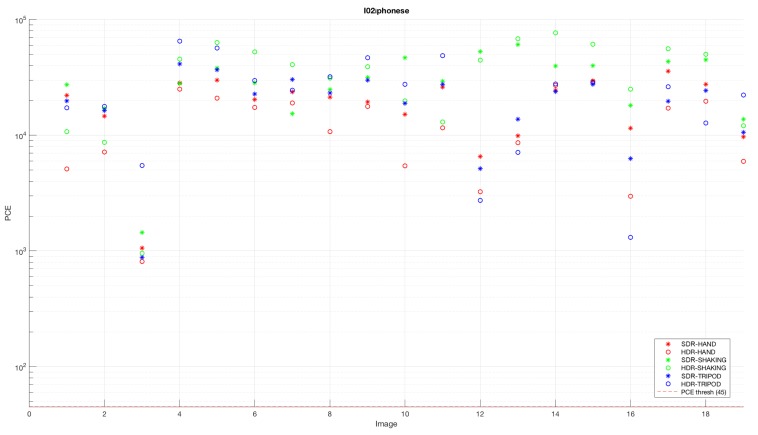
Example of the result obtained when correlating noise from HDR images captured by the I02 model with the SDR image fingerprint. I, iOS.

**Figure 8:**
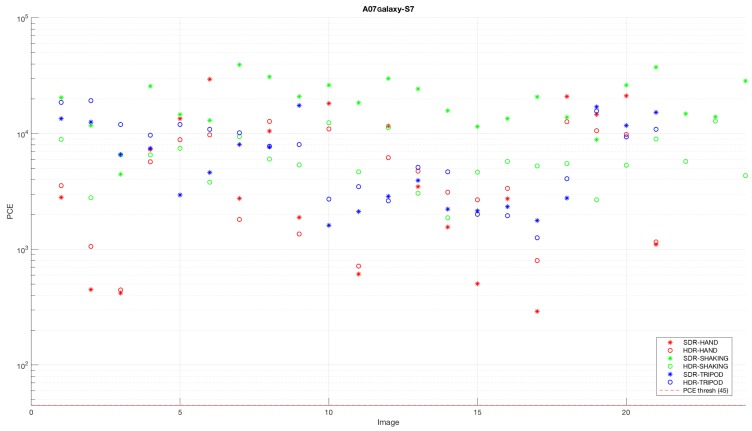
Example of the result obtained when correlating noise from SDR images captured by the A07 model with the HDR image fingerprint. A, Apple.

It is worth noting that the majority of SDR images combined with SDR-based fingerprints, as well as HDR images combined with HDR-based fingerprints produced PCE values higher than the threshold value. On the other hand, the combination of different types of images and fingerprints resulted in varying PCE values, depending on the employed camera device. An example of the results obtained by a single device is shown in Figure 7. It is noticeable that the PCE values of the HDR images captured by the I02 device model were above the threshold value for all the tested images, when they were correlated with the fingerprint of the SDR image set. This occurred regardless of camera movements. Similar results were obtained by using SDR images captured by a completely different device model, A07, and correlating them to the fingerprint of HDR images. The obtained result is shown in Figure 8. The analysis results led us to the conclusion that some of the devices can be identified more easily than other, and that the correct identification of those devices can be provided regardless of the type (HDR or SDR) of images. On the other hand, most of the devices have shown significantly different PCE values of images, depending on their type.

### 5.2. Analysis in Terms of Fingerprint Type

As it was concluded that, in most cases, the combination of different types of images and one fingerprint type gave a lower PCE value in comparison to the case of employing only SDRs or HDRs in the analysis, it is useful to analyze the impact of different fingerprints on the same sets of images. Comparing Figure 5 and Figure 6, it is noticeable that SDR images had a higher PCE value when the HDR-based fingerprint is employed in the case of A01, A05, I01 and I03, for each of the motion scenarios. A difference in terms of PCE values between scenarios cannot be seen for these devices. On the other hand, images taken by Devices A11, A15 and A17 had a significantly higher PCE value when the correlation of SDRs was computed with the SDR-based fingerprint. Images captured by all of the other devices are shown to have similar PCE values for both of the fingerprints.

The analysis of fingerprint impact on PCE values was performed for HDR images, as well. Images from Devices A11 and A15 were shown to have higher PCE values in correlation with the SDR-based fingerprint. While A11 showed no differences in the amount of PCE improvement for different motion scenarios, images from A15 had a significantly higher PCE in the case of capturing by a shaky hand. Improvements were noticeable in the case of images captured using a tripod, but there were no differences in the case of images taken by a steady hand. In contrast to the previously-mentioned devices, A01, A17, I01 and I03 showed better performances when their HDR images were correlated with the HDR-based fingerprint. Differences in terms of camera motions were not noticeable in these cases.

The obtained results for Devices A13 and A14 were especially interesting, because they showed a distinctive difference between SDR and HDR images taken by those devices. SDR images were shown to have high PCE values, no matter if the correlation were performed using the flat SDR- or HDR-based fingerprint, while HDR images obtained low PCE values, except from the images captured while shaking the camera device. Therefore, fingerprint type did not have a big influence on the results in this case, but the type of images did.

### 5.3. MIX Category Results’ Analysis

**Figure 9:**
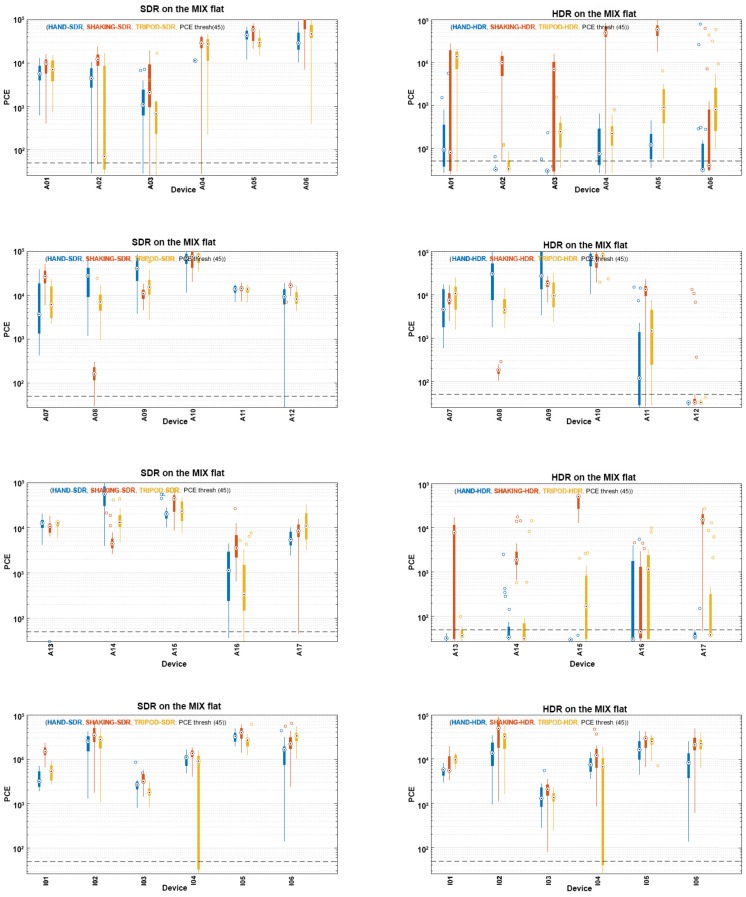
PCE values obtained by SDR and HDR images when compared with a flat MIX-based fingerprint.

The previously described analysis has shown that sources were, very often, identifiable in the cases when correlation was computed between the HDR image noise and SDR image fingerprint and vice versa. Considering this fact, it is natural that fingerprints computed from the MIX FLAT set of images provided good results, as well. They are shown in Figure 9. As the MIX category includes both HDR and SDR images, it was considered to be the most relevant for the analysis.

The averaged PCE of images captured by most of the devices was above the threshold when the MIX category was used as a reference, as can be seen from Figure 9. SDR images from Devices A01–A06 showed better performances than their HDR counterparts when they were captured by hand and a tripod. Images captured by a shaking hand using Devices A02, A04 and A05 were shown to have similar PCE values, regardless of the image type, in correlation with the MIX flat fingerprint. The other half of the devices from this set had shown better results for SDR images in the case of shaking motion. Captures made by a shaky hand led to bigger variations in the results, compared to more steadily captured images. This observation was expected and can be explained by the fact that the camera movement shifted the fingerprint matrices, making different offsets for the analyzed images. The offset depended on the velocity of the camera, which had not been measured during the dataset formation process.

The difference between SDRs and HDRs in terms of the PCE value was not significant for Devices A07–A10 when the MIX flat fingerprint was employed. SDRs have shown better performances for Devices A11 and A12, with the exception of images taken by a shaky hand using A11. In that case, the PCEs were comparable for both SDRs and HDRs. Similar conclusions can be made by analyzing the results obtained for Devices A13–A17, where only captures in shaking motion taken by Devices A15 and A17 have similar PCE values for both SDR and HDR images, while the rest of the devices and scenarios show the advantage of SDR images in the source identification process using the PRNU method.

Deviation from the previous results occurred in the analysis of iPhone devices. Images taken by I01–I06 with different motions have shown comparable PCE values for both SDR and HDR images. All the values were above the threshold, with the exception of the part of the images taken by I04 using the tripod. These results led us to the conclusion that iPhone devices are more easily identifiable image sources than other devices included in this research, no matter the type of analyzed image. This conclusion corresponds to the one conducted after analyzing the impact of using different types of images and the same SDR- or HDR-based fingerprint for PRNU computation.

### 5.4. Reliability of Source Identification

The threshold value was chosen to be equal to 45. Results have shown that both SDR and HDR image sources can be detected using this value, with the exception of HDR images taken from Devices A12, A14 and partially A6 and A17. Considering this fact, it is clear that the PRNU method cannot be generally applied, because the devices themselves can introduce variable hidden digital content to the images they produce or affect the procedure in another manner.

The most reliable source identification was made for Devices A07, A09, A10, I01, I02, I03, I05 and I06. Camera movements and usage of flat images were shown to have a minimal effect on the PCE value for the previously mentioned devices. Images taken by iPhone devices were shown to have a PCE value above the threshold and therefore provided digital source identification using the PRNU method. On the other hand, Devices A06, A12 and A16 were shown to give better PCE values for SDR, than HDR images. Furthermore, source identification from SDR images was less prone to camera movements for those devices. Taking the previous statements into account, it can be concluded that the complexity of HDR images introduces difficulties in digital source identification for some devices. This phenomenon requires further analysis of the HDR images creation procedure for the devices of interest.

### 5.5. Analysis of Low PCE Values

**Figure 10:**
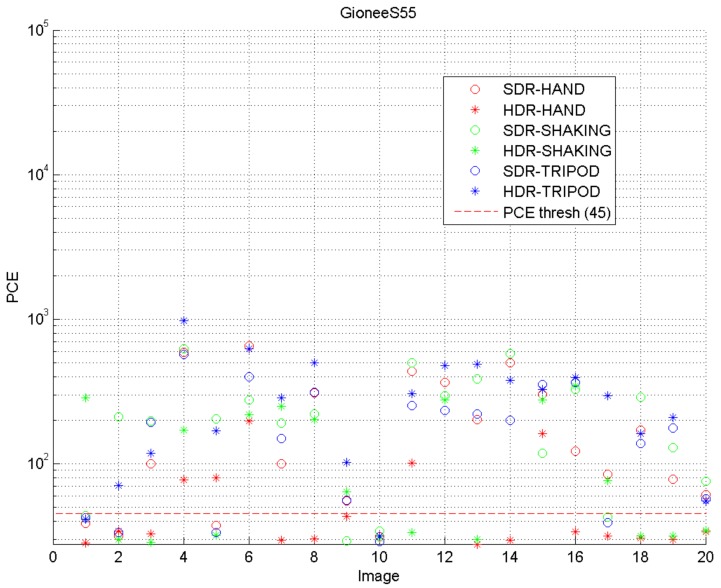
Example of the result obtained correlating noise from HDR images captured by A01 with the SDR image fingerprint.

The result obtained from A01 is provided in Figure 10. Twenty groups of images were captured in different motions and modes. Each group was provided the same image content as the controlled variable. Considering the difference of acquisition processes of SDR and HDR images, it can be concluded, by comparing the PCE values among three different motions, that image alignment had a serious impact on the performance of the PRNU-based method. As shown in Figure 10, the PCE values of SDR images were higher than the ones of HDR images captured with the hand motion. However, situation is opposite with the tripod motion. The reason could be that the image alignment operation with the hand motion changed the positions of pixels, which led to the mismatch between the noise image extracted from the HDR image and R-PRNU. In the case of the tripod motion, multiple images with perfect alignment were used to extract the noise image. It is well known that the more images are employed, the more precisely is the PRNU calculated. Therefore, higher PCE values would be obtained for HDR images in this case. In the case of the shaking motion, depending on the algorithms used in each device, on the one hand, the shift among the images would be too big to align them, which improved the PCE value of HDR images. On the other hand, image alignment was executed, reducing the PCE value of HDR images.

**Figure 11:**
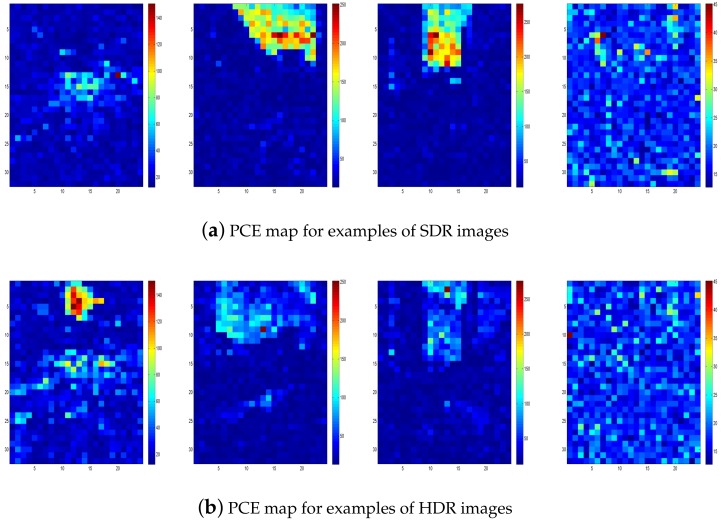
PCE maps for examples of SDR and HDR images.

In order to further explore the reason behind the change of PCE value between SDR and HDR images, the PRNU method based on the pixel patches was applied. Firstly, the images and R-PRNU were cropped into non-overlapping pixel patches with a 128 × 128 size. Then, the PCE values for each pixel patch were calculated, and for each image pair (SDR and HDR images), they were mapped into the same scale with the log function, in order to obtain the PCE map. PCE maps of SDR and HDR images given in Figure 1 captured with the hand motion are shown in Figure 11. An interesting phenomenon occurred at smooth image regions with low luminosity, such as the ground with low brightness, where PCE values of HDR images had higher values than their SDR counterparts. The same results were obtained for both over- and under-exposed image regions. On the contrary, PCE values were decreased for the pixel patches with smooth and high luminance, such as the blue sky. The reason could be that HDR images kept a balance between the dark and bright areas, and the PRNU-based method performed better for the images with much smoother and higher luminance. According to the above analysis, we can make the conclusion that for the smooth pixel patches with higher luminance, but not saturation, HDR and SDR images both had high PCE values. Moreover, image regions with over-/under-exposure usually led to a low PCE value. In addition, the images captured with a strong amount of noise, such as the night scene shown in the last column of Figure 11, also had a low PCE value.

The above presented analysis is more specific, rather than general, due to the fact that each device has its own specifics, which directly influence the results of PCE values obtained on images acquired by them. Considering that, further analysis in terms of image acquisition [34] and sensor pattern noise specifics [35] is required. The proposed dataset provides the ability for this and wider research, such as estimation of displacement fields from pairs of digital images [36] and characterization of the dynamic behavior of a mechanical chain tensioner by functional tolerancing [37].

## 6. Conclusions

With the help of powerful editing software programs, digital media has become vulnerable to manipulation. One of the possibilities of coping with this problem is digital source identification. This process is challenging, especially for non-standard images, such as HDRs, due to the complexity of their creation and wider dynamic range.

In this paper, we have presented a novel image dataset composed of both SDR and HDR images captured by several smartphone devices. The dataset has been built under controlled acquisition conditions, ensuring that it can be used by the image forensic community for several applications. As an example of using the dataset, this paper represented source identification performed using reference pattern noise, by employing PRNU matching. The analysis has shown that standard SDR images provide more reliable source identification in comparison to HDR images when the PRNU method is applied. Out of the total of seven brands of employed devices, only one brand has shown very small differences in the results for SDR and HDR images, which implies that source identification depends on the device characteristics themselves. This fact can serve as a motivation for the analysis of the acquisition processes adopted by each device. Research focusing on this topic can provide valuable results for the digital source identification process, due to the fact that hardware components leave their marks during the acquisition stage, thus producing a unique camera fingerprint.

The types of single images, as well as the types of images used for fingerprint computation were shown to have an impact on the obtained PCE values used for identification purposes. Moreover, examination of the effect of camera motions at the moment of capturing has shown that motions have a bigger impact on source identification in the case of HDR images, compared to SDR images. Differences in the results were less noticeable in the case of images captured by a steady camera, although they were generally dependent on the device type.

Despite difficulties in processing HDR images and identifying the source camera, the PRNU algorithm has shown its robustness, enabling correct source identification for a large number of tested devices. However, the novel dataset introduced in this paper can be employed in research on the topic of improving the performances of source identification based on HDR images.

## Figures and Tables

**Table 1:** Characteristics of the employed devices and captured images.

Device Class	Device Name	Brand	Model	OS	Image Resolution	SDR Flat	HDR Flat	SDR Hand	HDR Hand	SDR Shaking	HDR Shaking	SDR Tripod	HDR Tripod
A12	Huawei-Honor6plus	Huawei	PE-TL10	Android 6.0	2448 × 3264	50 (wall)	50 (wall)	20	20	20	20	20	20
A13	Huawei-Honor6plus	Huawei	PE-TL20	Android 4.4.2	2448 × 3264	50 (wall)	50 (wall)	20	20	20	20	20	20
A02	Huawei-P8	Huawei	GRA-L09	Android 6.0	4160 × 3120	50 (wall)	50 (wall)	24	24	24	24	24	24
A06	Huawei-Y5	Huawei	CUN-L21	Android 5.1	3264 × 2448	50 (wall)	50 (wall)	24	24	24	24	24	24
A04	Huawei-P10	Huawei	VTR-AL00	Android 7.0	3968 × 2976	51 (wall)	50 (wall)	15	15	20	20	26	28
A03	Huawei-Honor9	Huawei	STF-AL00	Android 7.0	3264 × 1840	50 (sky)	50 (sky)	20	20	20	20	20	20
A05	Huawei-Mate10Pro	Huawei	BLA-L29	Android 8.0	3968 × 2976	50 (wall)	50 (wall)	24	24	24	24	24	24
A09	Galaxy-Note5	Samsung	SM-N920C	Android 7.0	5312 × 2988	50 (sky)	50 (sky)	24	24	24	24	24	24
A07	Galaxy-S7	Samsung	SM-G930F	Android 7.0	4032 × 3024	52 (wall)	50 (wall)	21	21	24	24	21	21
A08	Galaxy-S7	Samsung	SM-G930F	Android 7.0	4032 × 2268	50 (sky)	50 (sky)	24	24	24	24	24	24
A10	Galaxy-J7	Samsung	SM-J730F	Android 7.0	4128 × 3096	50 (sky)	50 (sky)	24	24	24	24	24	24
A15	Xiaom-3	Xiaomi	Redmi Note3	Android 7.1	4608 × 2592	50 (wall)	50 (wall)	24	24	24	24	24	24
A11	Xiaomi5	Xiaomi	MI 5	Android 7.0	3456 × 4608	50 (wall)	87 (wall)	21	21	21	21	21	21
A14	Xiaomi-5A	Xiaomi	Note 5A Prime	Android 7.1	4160 × 2340	50 (sky)	50 (sky)	24	24	24	24	24	24
A01	GioneeS55	Gionee	GN9000	Android 4.4	3120 × 4208	50 (sky)	50 (sky)	20	20	20	20	20	20
A17	AsusZenfone-2	Asus	ASUS_Z00ED	Android 6.1	3264 × 1836	50 (sky)	50 (sky)	24	24	24	24	24	24
A16	OnePlus-3t	OnePlus	A3003	Android 8.0	4640 × 3480	50 (wall)	50 (wall)	24	24	24	24	24	24
I06	iPhone 5S	Apple	15A372	iOS 11	3264 × 2448	50 (wall)	50 (wall)	24	24	24	24	24	24
I04	iPad Air	Apple	A1475	iOS 11.0.1	2592 × 1936	50 (wall)	50 (wall)	24	24	24	24	24	24
I05	iPhone 6	Apple	A1586	iOS 11.3	2448 × 3264	50 (wall)	50 (wall)	21	21	21	24	21	21
I02	iPhone se	Apple	A1723	iOS 10.3.3	4032 × 3024	54 (sky)	54 (sky)	19	19	19	19	19	19
I03	iPhone 7	Apple	A1778	iOS 11.3	4032 × 3024	50 (wall)	50 (wall)	24	24	24	24	24	24
I01	iPhone-8	Apple	A1863	iOS 11.3	3024 × 4032	50 (sky)	50 (sky)	15	15	15	15	15	15
